# Spatio-Temporal Dynamics of Cholera during the First Year of the Epidemic in Haiti

**DOI:** 10.1371/journal.pntd.0002145

**Published:** 2013-04-04

**Authors:** Jean Gaudart, Stanislas Rebaudet, Robert Barrais, Jacques Boncy, Benoit Faucher, Martine Piarroux, Roc Magloire, Gabriel Thimothe, Renaud Piarroux

**Affiliations:** 1 Aix-Marseille Université, UMR 912 SESSTIM (AMU, INSERM, IRD), Marseille, France; 2 University College London, Department of Statistical Science, WC1E 6BT, London, United Kingdom; 3 Aix-Marseille Université, UMD 3, Marseille, France; 4 Ministère de la Santé Publique et de la Population, Port-au-Prince, Haiti; University of Oklahoma Health Sciences Center, United States of America

## Abstract

**Background:**

In October 2010, cholera importation in Haiti triggered an epidemic that rapidly proved to be the world's largest epidemic of the seventh cholera pandemic. To establish effective control and elimination policies, strategies rely on the analysis of cholera dynamics. In this report, we describe the spatio-temporal dynamics of cholera and the associated environmental factors.

**Methodology/Principal findings:**

Cholera-associated morbidity and mortality data were prospectively collected at the commune level according to the World Health Organization standard definition. Attack and mortality rates were estimated and mapped to assess epidemic clusters and trends. The relationships between environmental factors were assessed at the commune level using multivariate analysis. The global attack and mortality rates were 488.9 cases/10,000 inhabitants and 6.24 deaths/10,000 inhabitants, respectively. Attack rates displayed a significantly high level of spatial heterogeneity (varying from 64.7 to 3070.9 per 10,000 inhabitants), thereby suggesting disparate outbreak processes. The epidemic course exhibited two principal outbreaks. The first outbreak (October 16, 2010–January 30, 2011) displayed a centrifugal spread of a damping wave that suddenly emerged from Mirebalais. The second outbreak began at the end of May 2011, concomitant with the onset of the rainy season, and displayed a highly fragmented epidemic pattern. Environmental factors (river and rice fields: p<0.003) played a role in disease dynamics exclusively during the early phases of the epidemic.

**Conclusion:**

Our findings demonstrate that the epidemic is still evolving, with a changing transmission pattern as time passes. Such an evolution could have hardly been anticipated, especially in a country struck by cholera for the first time. These results argue for the need for control measures involving intense efforts in rapid and exhaustive case tracking.

## Author Summary

Cholera is the prototypical “waterborne” disease that can provoke deadly acute watery diarrhea epidemics in settings deprived of clean water and proper sanitation. In spite chronic deprivation, Haiti had been spared cholera for a century until the vibrio was imported in October 2010, which triggered the largest national epidemic ever recorded. To better understand the progression of the epidemic and adapt control measures, we describe and analyze the spatio-temporal dynamics and underlying factors associated with the first year of this cholera epidemic in Haiti. Attack rates reached highly heterogeneous levels between communes (from 64.7 to 3070.9 cases per 10,000 inhabitants), thereby suggesting disparate outbreak processes. While the first principal outbreak spread centrifugally like a damping wave that suddenly emerged from Mirebalais and Lower Artibonite, a second principal outbreak erupted at the end of May 2011, concomitant with the rainy season, and displayed a highly fragmented epidemic pattern. Environmental factors, such as rivers and rice fields, appeared to play a role in disease dynamics exclusively during the beginning of the epidemic. The dynamics of the cholera epidemic varied from place to place as time passed, following no clearly predictable scheme. Therefore, cholera control measures in Haiti should include rapid and exhaustive case tracking.

## Introduction

Cholera appeared in Haiti in October 2010, probably for the first time in the country's history [1]. Importation of the vibrio [2], [3] triggered an epidemic that rapidly proved to be the world's largest epidemic of the seventh cholera pandemic. In January 2012, a cholera elimination objective was adopted by Haitian and Dominican authorities, the World Health Organization (WHO), the United Nations International Children's Emergency Fund (UNICEF), and many of their partners [4]. However, to establish effective control and elimination policies, strategies rely on the analysis of the dynamics of cholera dissemination. To bolster control policies, various mathematical models have been established [5]–[8]. They have provided varying results, thereby demonstrating the importance of mathematical assumptions and parameter estimations [9], [10]. One model, issued in March 2011, has predicted 779,000 cases and 11,000 deaths for November 2011 [5]. Another model has predicted that the principal peak of the epidemic would occur in April 2011 in several departments [6]. Other studies acknowledged that this peak occurred in December 2010 but predicted tens of thousands of cases for March and April 2011 [7], [8]. Among the various causes of inaccurate predictions, all reports have used observed cases at the departmental scale, which hardly exhibit outbreak dynamics. Andews *et al.* [5] have not explicitly modeled spatial diffusion, while other authors [7], [8] estimated parameters at the country level, assuming homogeneous dynamics between all locations [9]. In contrast, cholera epidemic curves provided during the year 2011 by the Haitian Ministry of Health and Population showed that the cholera evolution profiles greatly varied from that predicted by models, with a marked and unexpected reduction in cholera incidence during the first months of 2011 followed by a new outbreak in May. This observation reveal how crucial it is to generate a comprehensive description of cholera diffusion and monitor cases daily at a communal scale. In other areas affected by recurrent cholera epidemics, it has been shown that studying the spatio-temporal dynamics of cholera outbreaks helped to define more effective control procedures [11]–[15]. Currently, only one publication [16] describes data at this spatio-temporal scale; however, this report aimed only to understand the dynamics of the cholera epidemic during the initial weeks following the outbreak onset. Since this first phase of the epidemic, many other cases have been reported across Haiti with new peaks and possibly new patterns of transmission. Therefore, the objective of the present study was to describe the spatio-temporal dynamics of the first year of this cholera epidemic in Haiti, identify the principal factors explaining the heterogeneity, and assess the epidemic processes.

## Methods

### Cases and deaths

Cholera-associated morbidity and mortality data were prospectively and anonymously collected by the Departmental Health Directorates at the commune level. Departmental databases were sent to the Haitian Directorate of Health (*Laboratoire National de Santé Publique*, LNSP), where data were gathered and analyzed after quality control.

According to the WHO standard definition [17], a probable cholera case was defined as profuse acute watery diarrhea with severe dehydration. Bacteriological confirmation of cases was recurrently performed at the LNSP for samples collected throughout the entire country using standard methods [18]. The in-hospital case fatality rate (ICFR) was defined as the ratio of cumulative number of deaths reported at Cholera Treatment Centers (CTCs) to cumulative number of hospitalized cases (severe cases). The case fatality rate (CFR) was defined as the ratio of cumulative number of in-hospital deaths to cumulative number of cases (reported at any health structure). As some communes lacked proper health facilities, some cholera patients had to travel to health structures of the nearest commune. To avoid overestimating case numbers in such locations and underestimating case numbers in surrounding areas, the data derived from these neighboring communes were aggregated after interviewing local health actors and analyzing local reports.

In this study, we did not include personal medical data but included the number of incident cases anonymously reported at each health facility. This study was approved by the Haitian Ministry of Public Health and Population (*Ministère de la Santé Publique et de la Population*).

### Methods

First, the mapping of global attack rates, mortality rates, ICFRs and CFRs observed between October 16, 2010 and October 15, 2011 was performed to assess the spatial distribution of the epidemic. Spatial autocorrelation was estimated using Moran's I statistic for areal data [19].

Second, temporal observations for the entire country were assessed to define epidemic phases and trends. Phases were specified using main slope changes in time series after mobile average (MA) smoothing (order two). The accuracy of this phase specification was then assessed by using sensitive analysis of the MA order, concordance with the wavelet analysis (see above), and the field expertise of the Haitian epidemiologists. For each epidemic phase, communal daily incidence rates (DIRs) were mapped, and spatial clustering was assessed using Kulldorff statistic [20]. To detect high-risk spatial clusters of cases, this algorithm moves a circular (or elliptic) scanning window over the study region, centered on each communal centroid with a radius ranging from 1% to 50% of the population at risk. This algorithm compares observed and expected case numbers inside and outside each window and estimates risk ratios based on the Poisson distribution. Using circular scanning windows, cluster significance (p-value) was calculated with a likelihood ratio test using the Monte Carlo approach with 999 random simulations under the null hypothesis of no clustering [19], [21]. Communal epidemic profiles of the different epidemic phases were compared and classified using hierarchical cluster analysis (HCA) based on Euclidean distance [22], and profile classes were then mapped. HCA is an unsupervised classification method that groups similar observations (the epidemiological curves for each commune) into classes depending on a similarity criterion (the daily case numbers recorded for each commune). Furthermore, to address the impact of population immunity, we assessed the impact of the accumulation of cases during the second outbreak. We compared the influence of cumulative incidences (aggregating phases 1 to 4) with the incidences observed during the second epidemic (phases 5 to 6) at the commune level using the Spearman correlation coefficient.

Third, to assess the environmental factors associated with outbreak spread, cases and rainfall time series at the country level were analyzed using wavelet spectrum analysis. By reducing the noise and capturing the local behavior of non-stationary time series [23], this approach detect underlying phenomena [24], [25], such as periodic variations, regime shifts or sudden perturbations and jumps. This method provides a multiscale analysis extracting the main evolution and trends of time series at different temporal scales and has been previously utilized to study cholera outbreaks [26], [27]. The relationship between cases and rainfall time series was assessed via cross-spectrum analysis [28]. Daily accumulated rainfall data were obtained from NASA Goddard Earth Sciences. These observations (TMPA-RT 3B42RT) were derived from the Tropical Rainfall Measuring Mission (see http://disc.sci.gsfc.nasa.gov/giovanni/overview/index.html for details). For each epidemic phase at the commune level, we also examined the relationship between cases and the following land cover surface factors: plains, mountains and hills, urban zones, rice fields, length of perennial rivers (10 km), area (km^2^), and number of watersheds, which were obtained from the MULTI-MENACES-HA team report [29]. These environmental factors were assessed via multivariate analysis using the Generalized Additive Model (GAM) derived from linear regression models [30], [31]. Standardized incidence ratios (SIRs) were estimated using log-transformed population density (as an offset variable) and were adjusted on the spatial distribution of communes modeled by thin plate splines following Wood's approach [30]. Because of the over-dispersion of cholera incidences, several models of the Negative Binomial and the Poisson families [32] were first graphically verified to meet the conditions of use and then compared using the Generalized Cross-Validation (GCV) score and the Un-Biased Risk Estimator (UBRE) score [30]. The stepwise selection of variables was performed using the GCV and UBRE scores. The explained deviance was also verified for model goodness-of-fit assessment. The SIRs and corresponding 95% confidence intervals (95CI) were estimated using the final selected model and tested.

Spatial cluster analyses were performed using SaTScan® v8.2.1 (Martin Kulldorff, Harvard Medical School, Boston, MA, USA and Information Management Services Inc, Silver Spring, MD, USA). Wavelet spectrum analyses were performed using Matlab® v7.1 (The Mathworks Inc., Natick, MA, USA). The other statistical analyses were performed using R® v2.13.0 (The R Foundation for Statistical Computing, Vienna, Austria) with *mgcv* package (GAM modeling), the *DCluster* package (spatial analysis), and the *cluster* package (HCA). The p-values were compared with the probability threshold α = 0.05. The maps were generated using Quantum-GIS® v1.7.3 (Open Source Geospatial Foundation Project, Beaverton, OR, USA).

## Results

One year after October 16, 2010, 493,069 cases and 6,293 deaths associated with cholera had already been reported in Haiti. The global attack rate was 488.9 cases per 10,000 inhabitants, and the global mortality rate was 6.24/10,000 inhabitants. The global ICFR and CFR were 1.76% and 0.83%, respectively. During this first year, 852 of the 1,437 stool specimens collected in the ten departments of Haiti were positive for *Vibrio cholerae* O1 Biotype El Tor, serotype Ogawa. No switch to the Inaba serotype was observed until the second year of the epidemic.

**Figure 1:**
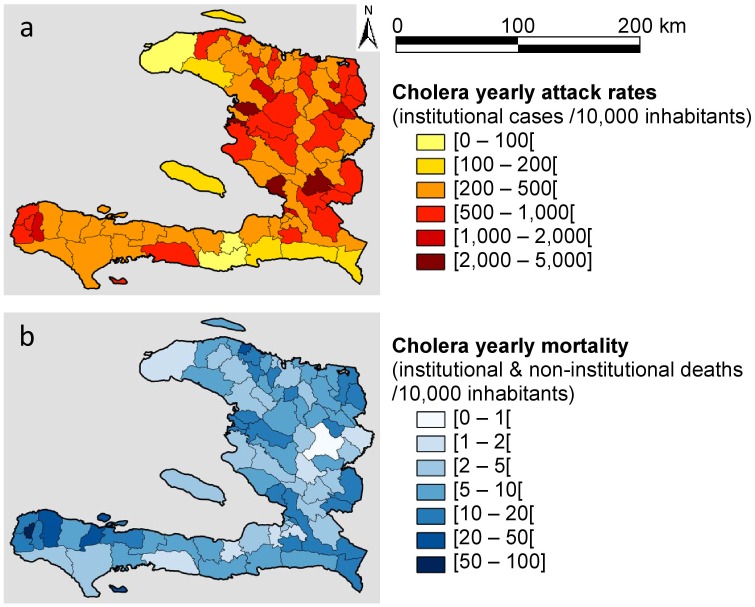
Mapping one year of cholera morbidity and mortality rates in Haiti. The colored scales represent yearly attack (a) and mortality (b) rates per 10,000 inhabitants in communes of Haiti (from October 16, 2010 to October 15, 2011).

The mapping of yearly attack rates (Figure 1a) showed that communes were disparately affected, as the rates ranged from 3,070.9 cases/10,000 inhabitants in Mirebalais (Department of Centre) to 64.7 cases/10,000 inhabitants in the western tip of the north peninsula (communes of Baie de Henne, Bombardopolis, Jean Rabel, and Mole St Nicolas). The Moran's I coefficient was particularly low (I = 0.02, p = 0.5), thereby indicating no significant spatial autocorrelation and confirming the highly fragmented pattern at this scale. The mapping of mortality (Figure 1b) displayed high yearly mortality rates in the western tip of the south peninsula with 58.5 and 45.1 deaths/10,000 inhabitants in Chambellan and Pestel, respectively. The low spatial autocorrelation together with the high degree of spatial heterogeneity of incident cases showed that outbreak dynamics in Haiti varied from location to location. This fragmented spatial pattern drew attention to the need for separate analyzes at each phase of the outbreak, both at the country and local levels.

**Figure 2:**
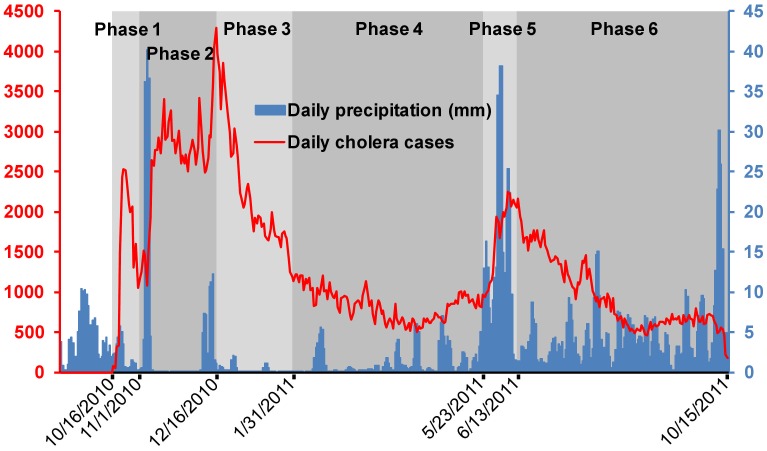
Temporal cholera dynamics. Daily cholera cases (red), daily rainfall (blue), and epidemic phases (grey) (September 15, 2010 to October 16, 2011) are presented. Accumulated rainfall data were obtained from the Daily Global and Regional Rainfall (TMPA-RT 3B42RT derived).

At the country level (Figure 2), the epidemic course exhibited two principal outbreaks.

Studying the slope changes, the time series were divided into two periods separated by the main peak of the cholera epidemic (12/16/2011, 4,289 cases). The period preceding this peak was split into two parts separated by the nadir in cholera cases occurring on October 31, 2010 (1,053 cases), which was observed just before the violent increase in cholera cases reported in early November. The period succeeding the principal peak was split into four parts. During the first part (phase 3), the attack rate dramatically decreased (from 3,972 to 1,131 cases daily). Phase 3 ended on January 30, 2011 and was followed by a lull period characterized by a reciprocation of small increases and decreases (phase 4), with an average of 835 daily cases (standard deviation SD = 188 cases) until May 22, 2011. On May 22, the slope of the epidemic curve changed to a marked increase, thereby signalling the onset of a new epidemic wave and the beginning of phase 5. After a high tray above 2,000 cases per day (until 06/12/2011), the final recorded decrease characterized phase 6.

**Figure 3:**
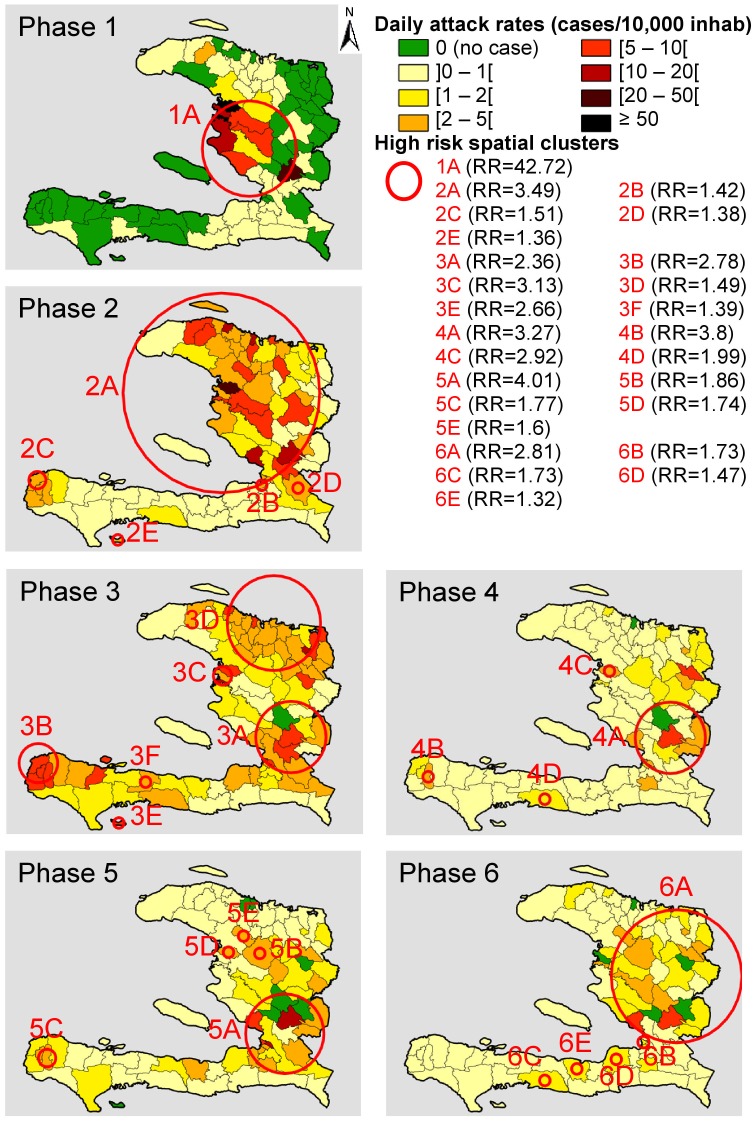
Daily incidence rates (DIRs) and high-risk spatial clusters for each epidemic phase.

The first principal outbreak started during a period with very little rainfall (∼2 mm/day during the last 15 days of October 2010). Outbreak onset lasted from mid- to late October (phase 1) and was associated with the introduction of *Vibrio cholerae* in Meille (commune of Mirebalais, Department of Centre) and the abrupt contamination of the Artibonite River [2],[16]. During this first phase, 23,587 cases were reported (DIR = 1.46 cases/10,000 inhabitants/day, 95CI[1.44–1.48]) (Figure 3). Spatial cluster analysis displayed only one significant high-risk cluster centered at the Artibonite Valley, with a significantly elevated relative risk (RR) of 42.72, 95CI[41.1–44.4] compared with the other regions of the country (p = 0.001), thereby confirming the link between cholera and proximity to the Artibonite River during the beginning of the epidemic.

During the second phase (November 1–December 15, 2010), cholera diffused out of the Artibonite Valley concomitant with Hurricane Tomas, and 119,347 cases were reported (DIR = 2.63 cases/10,000 inhabitants/day [2.62–2.65]). Among the five significant high-risk clusters, the largest cluster encompassed a large portion of the country including Port-au-Prince but spared the South Peninsula(RR = 3.49 [3.45–3.54], p = 0.001).

**Figure 4:**
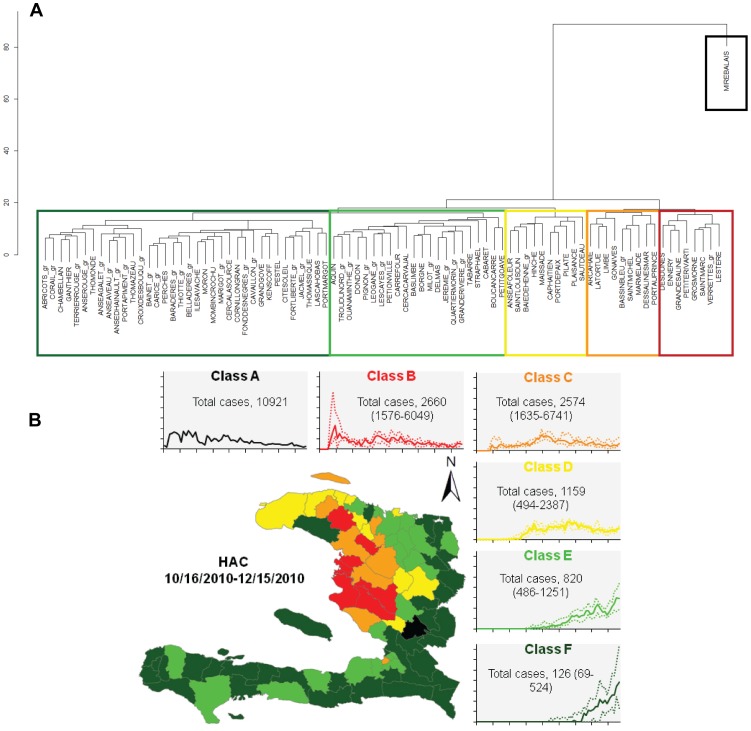
Epidemic profiles of the first outbreak phases (phases 1 and 2). a) Hierarchical cluster analysis (HCA) of communal epidemic profiles and b) Communal mapping of the epidemic profile classes. Median (25th–75th percentiles) communal cases observed during the period are provided for each class of profile. The graphs represent the median (solid line) and 25th–75th percentiles (dotted lines) of daily communal cases standardized by the total number of cases during the period.

The hierarchical cluster analysis (HCA) of these first two phases of the epidemic profiles (Figure 4) identified the outbreak origin in Mirebalais (Class A), where cases occurred primarily during the first month (DIR = 19.81 cases/10,000 inhabitants/day [19.44–20.18]). The class B profile was primarily located in the low Artibonite valley, where the outbreak began a few days later (DIR = 6.22 cases/10,000 inhabitants/day [6.18–6.26]). In this area, most cases occurred at the beginning of the wave, and then the number of daily cases decreased. Classes C and D (DIRs = 3.41 cases/10,000 inhabitants/day [3.0–3.46] and 3.05 cases/10,000 inhabitants/day [3.01–3.09], respectively) displayed a smoother pattern after a delay of approximately one week. Finally, the communes of classes E and F (DIRs = 1.25 cases/10,000 inhabitants/day [1.24–1.27] and 0.83 cases/10,000 inhabitants/day [0.82–0.85], respectively) were the last affected areas. Overall, the mapping of epidemic profile classes exhibited a centrifugal spread from the Artibonite Valley: distant communes displayed delayed outbreak onsets, lower daily incidence rates, and delayed and smaller outbreak peaks.

With 104,784 reported cases (DIR = 2.26 cases/10,000 inhabitants/day [2.25–2.27]) from December 16, 2010 to January 30, 2011, phase 3 was characterized by a marked decrease that was observed in all communes but was more marked in urban communes. Six significant high-risk spatial clusters were identified; the main cluster was centered at the mountains of the Department of Centre with a RR of 2.36 [2.32–2.39] (p = 0.001). The subsequent forth phase was a lull period ending on May 22, 2011 with 93,474 reported cases (DIR = 0.83 cases/10,000 inhabitants/day [0.82–0.83]). During this lull phase, four significant clusters of elevated incidence rates persisted. The main cluster was again localized to the mountains of the Department of Centre with a RR of 3.27 [3.32–3.32] (p = 0.001). The remaining clusters displayed particularly local and brief outbreaks.

The second principal outbreak began at the end of May (phase 5), concomitant with the onset of the rainy season, which started late in 2011 and was associated with 35,356 cases (DIR = 1.67 cases/10,000 inhabitants/day [1.65–1.69]). This outbreak peaked on June 12. Five significant high incidence clusters were observed, the main cluster still remained localized to the mountains of the Department of Centre with a RR of 4.01 [3.92–4.1] (p = 0.001).

The subsequent decrease (phase 6) included 116,306 cases (DIR = 0.92 cases/10,000 inhabitants/day [0.86–0.98]). Five significant high incidence clusters were identified; the main cluster encompassed approximately five departments (North-East, North, Centre, Artibonite, and portion of the West department), with a RR of 2.81 [2.78–2.85] (p = 0.001). The remaining clusters were located at communes of the south peninsula with local outbreaks. The positive correlation (0.35, p = 0.001) between the two principal outbreaks suggests that population immunity did not play a major role in the cholera epidemic dynamics during the first year. The effect of immunity during this period may be concealed by spatial aggregation of the data at the communal level, population movement during the first weeks of the outbreak, and most notably environmental or social intra-communal determinants.

**Figure 5:**
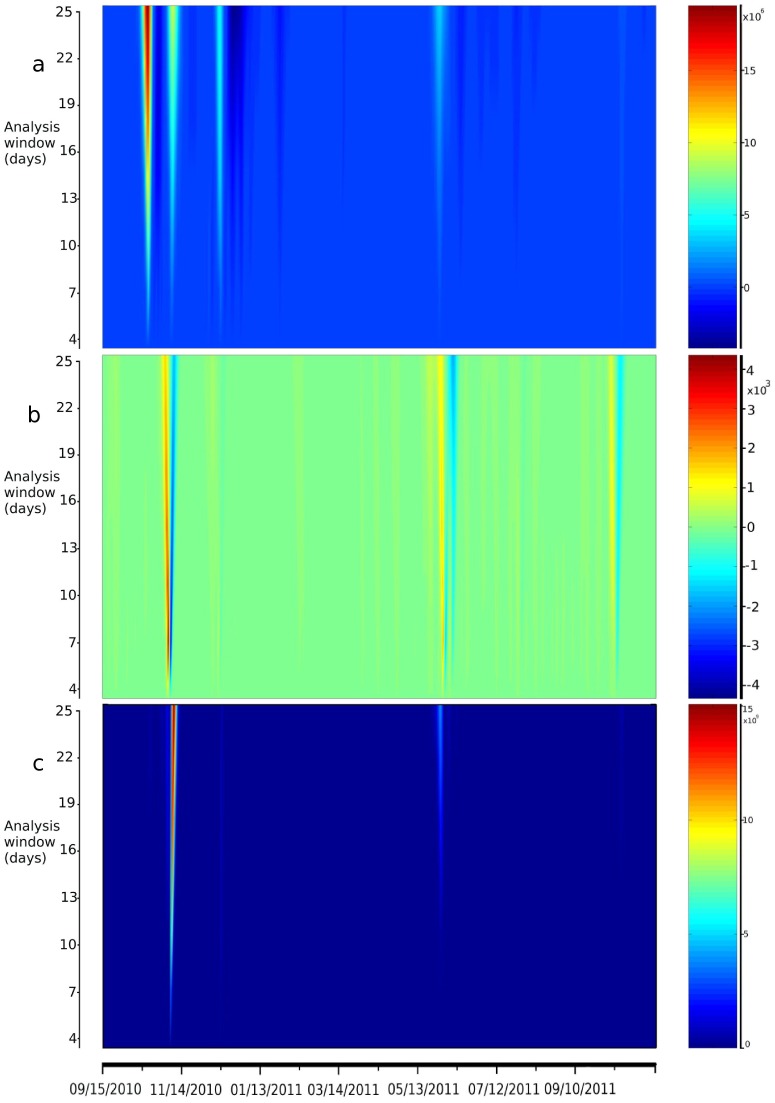
Spectral analysis of time series. Analysis of cases (a), rainfall (b), and cross-wavelet (c) between cases and rainfall are presented. The Y-axes represent length of the wavelet analysis window (from 3 to 26 days) and the color scales represent the spectral values for each length of the analysis window.

The patterns of these various phases were confirmed by spectral analysis of case time series (Figure 5a), which highlights the elevated velocity and intensity of the first phase in the Artibonite valley (phase 1) and the high (but less abrupt) intensity of phases 2 and 5. Spectral analysis of rainfall series (Figure 5b) highlighted the importance of rainfall during both Hurricane Tomas in November 2010 and the 2011 rainy season that began in May, which were the only two heavy rainfall periods associated with incident cases based on cross-spectrum analysis (Figure 5c).

Local environmental factors were assessed by quantifying their association with the spread of cholera at teach phase (Table 1). For phase 1, the results highlighted the role of the Artibonite River (Standardized Incidence Ratio for each 10 km portion of perennial rivers, SIR = 2.28 [1.86–2.79], p<0.001) and rice fields (SIR = 16.7 [3.0–93.7], p = 0.002). Conversely, urban zones (SIR = 0.034 [0.007–0.18], p<0.001) and mountainous zones (SIR = 0.113 [0.05–0.27], p<0.001) displayed a protective role. During phases 2, 4 and 6, no specific environmental factor was associated with outbreak spread. During phase 3, urban zones (SIR = 0.68 [0.47–0.97], p = 0.03) experienced a more rapid decrease in case numbers, thereby showing an apparent protective role. Other factors were no more significant during this phase. With the exception of phase 3, the spatial distribution of communes remained significant (p<0.006), thereby showing that environmental factors did not fully explain the spatial clustering of cases during each phase.

**Table 1:** Impact of local environmental factors during each epidemic phase.

	Phase 1: Oct 16, 2010 to Oct 31, 2010	Phase 2: Nov 1, 2010 to Dec 15, 2010	Phase 3: Dec 16, 2010 to Jan 30, 2011	Phase 4: Jan 31, 2011 to May 22, 2011	Phase 5: May 23, 2011 to Jun 12, 2011	Phase 6: Jun 13, 2011 to Oct 15, 2011
	SIR [95%CI] (p)					
Length of perennial rivers (10 km)	**2.28 [1.86–2.79] (<0.001)** †	-*	-*	-*	1.1 [1.0–1.21] (0.06)‡	-*
Number of watershed	-	-*	-*	-*	-*	-*
mountainous landscapes (vs. plains)	**0.113 [0.05–0.27] (<0.001)** †	-*	-*	-*	-*	-*
Urban zones	**0.034 [0.007–0.18] (<0.001)** †	-*	**0.68 [0.47–0.97] (0.03)** †	-*	-*	-*
Rice fields	**16.7 [3.0–93.7] (0.002)** †	-*	1.3 [0.95–1.8] (0.09)‡	-*	0.67 [0.43–1.06] (0.08)‡	-*
Spatial distribution of communes	**p<0.001** †	**p<0.001** †	-*	**p = 0.005** †	**p = 0.002** †	**p = 0.006** †

Standardized incidence ratios (p-values) were estimated using the multivariate regression model.

* Factor excluded using stepwise analysis.

† Significant factors (boldface).

‡ Non-significant factors kept using stepwise analysis.

## Discussion

With 493,069 cases after one year, the cholera epidemic in Haiti appear to be the largest ever recorded in a single country during the past 50 years. Although it began during the last trimester of 2010, the cases reported in Haiti accounted for more than 56% of the total cholera burden in 2010. Yearly attack rates were higher than 20% in several Haitian communes, such as Mirebalais (30.7%), L'Estere (29.2%), Grande Saline (22.1%), and Cabaret (26.6%). To make a comparison, the yearly attack rate during the 2008–2009 epidemic in Harare (Zimbabwe) was 1.29%, reaching a maximum in the Hopley suburb with 541 cholera cases per 5,994 inhabitants (9%) [33]. Before the Haitian epidemic, the largest cholera epidemic ever recorded during the seventh pandemic was the 1991 epidemic in Peru, which accounted for approximately 300,000 cumulative cases during the first year [34]. However, the yearly attack rate (approximately1.4%) of the Peruvian epidemic was approximately 3.5 times lower than that of the epidemic in Haiti (4.9%). Due to its exceptional amplitude, the cholera epidemic in Haiti led to a large number of fatalities. Because of the difficulties of identifying all cases and deaths in remote rural areas, it is likely that the recorded 6,293 deaths represent only a portion of the actual cholera death toll.

The analysis of epidemic profiles at different time phases reveals evidence of different spatio-temporal patterns. The first two phases of the epidemic (October 16–December 15, 2010) display a clear centrifugal expansion of cholera, with a damping wave centered at the location of the explosive outbreak onset in Mirebalais and the Artibonite Valley. Even if low rainfall had been recorded before early October, no heavy rain was associated with the outbreak onset (phase 1), and flooding cannot be incriminated. However, several environmental factors (rice fields, plains, rural zones, and rivers) were associated with a higher risk of contracting the disease during this early phase. These findings correlate with the results of previously published reports and studies that attribute the onset of the epidemic to massive contamination of the Artibonite River and downstream irrigation canals by an imported pathogenic strain of cholera [2], [16], [35].

Conversely, the particularly rapid diffusion of cholera out of the Artibonite Valley (November - mid-December 2010, phase 2) was not associated with any environmental factors but might be linked to other phenomena. Human-driven dissemination was favored by the massive contamination of the population living in the Artibonite Delta [16], the lack of immunity among Haitian population, and deficiencies in water, sanitation, and health care systems [35]. The explosive spread of the disease overwhelmed the humanitarian response and the initial attempts to broadcast awareness and hygiene messages. People who fled from the Artibonite Delta to neighboring communes [36] also played an aggravating role in cholera diffusion, thereby favoring the spread of cholera even in remote rural areas. The violent nature of this outbreak spread may also have been promoted by Hurricane Tomas, which reached Haiti on November 5 with rapid flooding in some areas already affected by cholera, such as Gonaives. Finally, riots in Port-au-Prince following the first round of presidential elections in early December 2010 may have also reinforced this explosive epidemic.

The relationship between rainfall and cholera spread in Haiti was attested by the association of phase 2 with Hurricane Tomas, the lull transmission period with the dry season (phases 3 and 4), and the second outbreak (phase 5) with the heavy rainfall during late May2011. This booster effect of rainfall on cholera outbreaks has been observed in many other countries [13], [14], [36]–[38], where rainfall has caused latrine overflow or the washing up of waste with subsequent contamination of wells and surface waters. However, the relationship between rainfall and cholera likely involves other mechanisms, such as the seasonal modification of human water sources or human behavior such as rice culture activity. Overall, many phenomena affecting environment-to-human and human-to-human transmission may affect this relationship between rainfall and cholera outbreaks, which therefore should not be regarded in Haiti as in Bangladesh, where cholera onset has been associated with vibrio blooms in aquatic reservoirs [39], [40].

During phases 3, 4 and 5, cholera incidence was poorly associated with environmental factors. Cholera attack rates decreased more rapidly in the main towns than in rural areas during phase 3. Sequential identification of spatial clusters during the successive phases of the epidemic shows that mountainous rural areas located in the northern and eastern portions of the country likely functioned as a reservoir for cholera during the dry season until more favorable climatic factors triggered the second outbreak of late May 2011. During a field assessment in April 2011, we found that cholera persisted during the lull period in rural Haiti, circulating from one village to another, and provoking outbreaks linked with the transient local contamination of springs and streams. Due to the difficulty in reaching these mountainous remote areas, the fight against cholera was less efficacious than in towns and plains. Unlike observations made in Asia, where cholera outbreak patterns largely depend on human exposure to the aquatic reservoirs of *V. cholerae* [41], or eastern Democratic Republic of the Congo, where lakes play an important role in outbreaks [13], [15], our results do not suggest any environmental persistence of cholera. However, this has to be confirmed with environmental sample studies. Currently, cholera presence in the environment has been reported in two cross-sectional studies [42], [43], but no environmental spatio-temporal monitoring system has been developed. In contrast, rice fields tended to be protective during the second outbreak (phase 5). This may be partly due to population immunity acquired during the initial phases of the epidemics, particularly in the Artibonite Delta, which was heavily stricken during the first phase of the epidemic. The protection was likely also due to the action of nongovernmental organizations (NGOs) and local actors as well as the reinforcement of a population sensitization program that was implemented in the Artibonite plain [44].

Overall, our findings clearly show that the epidemic is still evolving. Such diversity in transmission patterns could hardly have been anticipated, especially in a country struck by cholera for the first time, which highlights the need for comprehensive studies such as the current investigation. Therefore, we believe it is too early to predict the future pattern of this epidemic, and especially to affirm that cholera will become endemic in Haiti. Notably, the presence of estuaries in an area hit by cholera does not necessarily mean that *V. cholerae* will perennially settle in the brackish waters and that seasonal outbreaks will recurrently occur in the future. Madagascar, another island with deficient sanitation, a susceptible hydro-geologic environment, a widespread rice culture, political tension, and a lack of resources, was hit by successive cholera waves from 1999 to 2001 [45]. Since this time, the country has not experienced new outbreaks. Like Madagascar, Haiti may benefit from its insular position far from usual endemic foci. The current spatio-temporal analysis shows that dynamics of the cholera epidemic varied from location to location as time passed, following no clearly predictable scheme. Excluding the first phase, no recurrent environmental factor was implicated, except rainfall involved in the exacerbation of the epidemic. After the first phases of the outbreak, the absence of constant spatial clusters and the changing pattern of cholera distribution in Haiti argue for the need for control measures that should include intense efforts in rapid and exhaustive case tracking.
